# Datasets for testing the performances of jump diffusion models

**DOI:** 10.1016/j.dib.2016.11.014

**Published:** 2016-11-10

**Authors:** Weijun Xu, Guifang Liu, Hongyi Li

**Affiliations:** a School of Business Administration, South China University of Technology, Guangzhou 510640, China; b Business School, Chinese University of Hong Kong, Shatin, NT, Hong Kong

## Abstract

This article contains datasets related to the research article titled a novel jump diffusion model based on SGT distribution and its applications (”A novel jump diffusion model based on SGT distribution and its applications” (W.J. Xu, G.F. Liu, H.Y. Li, 2016) [1]). The datasets contain continuous composite daily percentage return values which are computed from the daily closing prices. Firstly, we describe statistical properties of the datasets. Then, the datasets are split into two samples, the in-sample data and out-of-sample data. The datasets can be used as benchmarks for testing the performances of jump diffusion models.

**Specifications Table**

**Table:** 

Subject area	*Economics*
More specific subject area	*Financial Engineering*
Type of data	*Table, figure, Excel file*
How data was acquired	*The datasets were acquired freely from the Wind Finance Database in China.*
Data format	*Raw*
Experimental factors	*In order to the empirical research, the dataset is split into two samples, the in-sample data and out-of-sample data.*
Experimental features	*The data is the daily percentage return values of four representative composite indices and is public data in financial market.*
Data source location	*Guangzhou, China*
Data accessibility	*Data is within this article (http://www.wind.com.cn/Default.aspx)*

**Value of the data**

• The data is convenient to execute the statistical analysis and empirical application in this paper.
• The data can be used to test the existence of jumps in four representative composite indices and estimate the relevant model parameters.
• The data can be used to assess the asset return distribution describing performance of relevant models.
• The data can be used to explore the volatility forecast performance of relevant models based on in-sample data and out-of-sample data respectively.

## 1 Data

• The raw data contains the daily closing price of four representative composite indices (the Nikkei 225 Index (NIKKEI225), the Dow Jones Industrial Average Index (DJIA), Hang Seng Composite Index (HSI), and the Shanghai Composite Index (SCI)). The time period is from January 3, 1995 to March 25, 2016.
• In order to explore the performance of jump diffusion models, the daily closing price is converted into daily percentage return value.

## 2 Experimental design, materials and methods

The datasets, daily closing price time series of asset $S_{t}(t=1,2,\ldots ,N)$, are obtained from the Wind Finance Database (http://www.wind.com.cn/Default.aspx) in China. In order to explore the asset return distribution describing performance of jump diffusion models, the datasets are converted into daily percentage return values $y_{t}$ by using the following equation:

$$y_{t}=100\times (\ln(S_{t})-\;\ln(S_{t-1}))$$

where $\ln(S_{t})$ is the natural logarithm of the closing price $S_{t}$ at t. All the datasets are listed in Table 1. The daily closing prices and daily percentage return values are shown in Supplementary materials (data.xlsx).

**Fig. 1:**
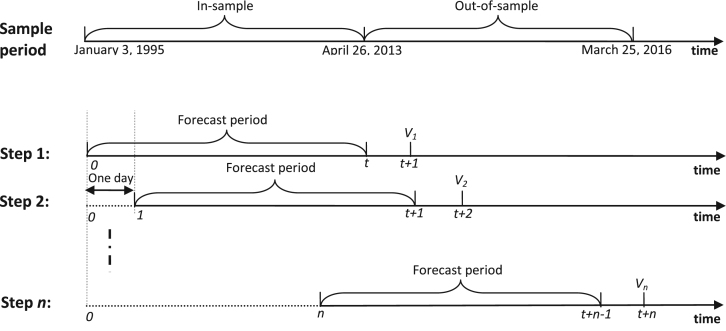
Scheme of the rolling time window used in the analysis. Notes: (0*,t*) is the initial time period of in-of-sample data; *V*_*n*_ is the forecast volatility which is obtained at step *n*.

Finally, on the performance of volatility forecasts, several GARCH family models with some compound return distributions are presented. The datasets are split into two samples, the in-sample data and out-of-sample data (see Fig. 1). In order to compare the performance of volatility forecasts of relevant models, we use the rolling-window approach (One step forward). The initial time period of in-of-sample data is from January 3, 1995 to 26 April, 2013. For each data series, these relevant models are first estimated using the in-of-sample data (before the time *t*), and a volatility value is obtained as a forecast volatility at the next time *t*+1 (see Fig. 1). Subsequently, the estimation period was rolled forward by adding one new day. By repeating this procedure, the out-of-sample volatility forecasts were calculated for the rest days.

## Figures and Tables

**Table 1:** Daily *percentage return values* datasets provided.

	**Dataset Name**	***N***	**Time interval**	**Country**	**Description**
**1**	NIKEEI225	5175	January 3, 1995–March 25, 2016	Japan	*Nikkei 225 Index*
**2**	DJIA	5196	January 3, 1995–March 25, 2016	USA	*Dow Jones Industrial Average Index*
**3**	HIS	5180	January 3, 1995–March 25, 2016	China	*Hang Seng Composite Index*
**4**	SCI	5146	January 3, 1995–March 25, 2016	China	*Shanghai Composite Index*
