# Supplementary material for: Datasets for testing the performances of jump diffusion models
Source: Data Brief. 2016 Nov 10;10:98–100. doi: 10.1016/j.dib.2016.11.014 (PMC5144646; doi:10.1016/j.dib.2016.11.014)
Supplement: Supplementary file 1 — Supplementary material [file mmc1.docx]

We declare that we have no conflict of interest.
